# A Novel User Utility Score for Diabetes Management Using Tailored Mobile Coaching: Secondary Analysis of a Randomized Controlled Trial

**DOI:** 10.2196/17573

**Published:** 2021-02-24

**Authors:** Min-Kyung Lee, Da Young Lee, Hong-Yup Ahn, Cheol-Young Park

**Affiliations:** 1 Division of Endocrinology and Metabolism, Department of Internal Medicine Myongji Hospital Hanyang University College of Medicine Gyeonggi-do Republic of Korea; 2 Division of Endocrinology and Metabolism Department of Internal Medicine Korea University College of Medicine Seoul Republic of Korea; 3 Department of Statistics Dongguk University-Seoul Seoul Republic of Korea; 4 Division of Endocrinology and Metabolism Department of Internal Medicine Kangbuk Samsung Hospital, Sungkyunkwan University School of Medicine Seoul Republic of Korea

**Keywords:** type 2 diabetes, mobile applications, diabetes management, patient engagement

## Abstract

**Background:**

Mobile health applications have been developed to support diabetes self-management, but their effectiveness could depend on patient engagement. Therefore, patient engagement must be examined through multifactorial tailored behavioral interventions from an individual perspective.

**Objective:**

This study aims to evaluate the usefulness of a novel user utility score (UUS) as a tool to measure patient engagement by using a mobile health application for diabetes management.

**Methods:**

We conducted a subanalysis of results from a 12-month randomized controlled trial of a tailored mobile coaching (TMC) system among insurance policyholders with type 2 diabetes. UUS was calculated as the sum of the scores for 4 major core components (range 0-8): frequency of self-monitoring blood glucose testing, dietary and exercise records, and message reading rate. We explored the association between UUS for the first 3 months and glycemic control over 12 months. In addition, we investigated the relationship of UUS with blood pressure, lipid profile, and self-report scales assessing diabetes self-management.

**Results:**

We divided 72 participants into 2 groups based on UUS for the first 3 months: UUS:0-4 (n=38) and UUS:5-8 (n=34). There was a significant between-group difference in glycated hemoglobin test (HbA_1c_) levels for the 12-months study period (*P*=.011). The HbA_1c_ decrement at 12 months in the UUS:5-8 group was greater than that of the UUS:0-4 group [–0.92 (SD 1.24%) vs –0.33 (SD 0.80%); *P*=.049]. After adjusting for confounding factors, UUS was significantly associated with changes in HbA_1c_ at 3, 6, and 12 months; the regression coefficients were –0.113 (SD 0.040; *P*=.006), –0.143 (SD 0.045; *P*=.002), and –0.136 (SD 0.052; *P*=.011), respectively. Change differences in other health outcomes between the 2 groups were not observed throughout a 12-month follow-up.

**Conclusions:**

UUS as a measure of patient engagement was associated with changes in HbA_1c_ over the study period of the TMC system and could be used to predict improved glycemic control in diabetes self-management through mobile health interventions.

**Trial Registration:**

ClinicalTrial.gov NCT03033407; https://clinicaltrials.gov/ct2/show/NCT03033407

## Introduction

### Background

The rate of diabetes has been steadily increasing over the past few decades [1,2], and its associated complications are major causes of morbidity and mortality [3] that lead to substantial economic loss through direct medical costs [4]. To prevent diabetes complications and decrease economic burden, multifaceted professional interventions are needed [5,6]. Successful treatment of diabetes includes patient self-management such as lifestyle intervention [7,8]. Clinical trials have shown that effective lifestyle modifications can substantially reduce the risk of developing diabetes and improve patient health outcomes [9,10].

Digital health technology-based tools have been developed to assist in diabetes self-management [11]. Due to increasing evidence for the efficacy of digital health tools for improving glycated hemoglobin (HbA_1c_) levels and other diabetes-related outcomes, both the 2017 National Standards for Diabetes Self-management Education and Support [12] and the 2019 American Diabetes Association Standards of Medical Care [13] recommend including technology-based solutions to deliver diabetes care and education. Mobile platforms and health applications are increasingly being implemented as useful tools for patients and health care providers [14] and play a role in supporting diabetes self-management by sharing data with providers and providing minimal data analysis, interpretation, and guidance to patients [15]. However, the effectiveness of digital health tools to improve diabetes outcomes could depend on patient engagement in the beginning, such as proper blood glucose testing, medication adherence, adoption of a healthy diet and physical activity, and advice-sharing text messages [16]. Patient engagement is increasingly regarded as a crucial factor in diabetes management [17]. Therefore, patient engagement must be examined through multifactorial tailored behavioral interventions because of the variability in self-management capability.

A 12-month randomized clinical trial demonstrating the effectiveness of tailored mobile coaching (TMC) on diabetes management among policyholders with type 2 diabetes was previously reported [18]. TMC is a mobile health care system in which the intensive coaching from health care providers and the self-application of patients are organically connected. The effectiveness of the TMC system can vary depending on patient engagement in diabetes self-management. In this study, we conduct a subanalysis of the TMC study to evaluate the relationship between patient engagement and diabetes-related health outcomes. We developed a novel user utility score (UUS) consisting of 4 major components of blood glucose testing [19], dietary habits [20], exercise [21], and message reading [22] as a tool to measure patient engagement.

### Objectives

This study examines the usefulness of UUS as a tool to measure patient engagement by using a mobile health application for diabetes management among policyholders with type 2 diabetes. The primary aim of this paper is to determine whether UUS for the first 3 months results in improved glycemic control over a 12-month follow-up period among policyholders with type 2 diabetes. In addition, we investigate the relationship of UUS with blood pressure, lipid profile, and diabetes self-management.

## Methods

### Ethics Approval and Consent to Participate

All participants provided written informed consent before any study procedures were started. The trial protocol was reviewed and approved by the institutional review board of Kangbuk Samsung Hospital (KBS12089) and was conducted in accordance with the Helsinki Declaration of 1975.

### Study Population and Design

This study was conducted with Korean policyholders with type 2 diabetes recruited from Samsung Fire and Marine Insurance (Seoul, South Korea) from October 2014 to December 2015. This study was an open-label, randomized controlled trial to evaluate the effectiveness of the TMC system provided by Kangbuk Samsung Hospital, Seoul, Korea. Participants were randomly assigned into an intervention group and a control group. During a 6-month assessment period, the intervention group received TMC for diabetes management via a mobile app, whereas the control group maintained their usual diabetes care. After 6 months, the second 6-month period of the study was conducted and included the subjects who agreed to participate. Identification and recruitment of patients have been described in the previous study [18].

We conducted a subanalysis of results from the TMC group over 12 months. During the first 6-month period, 72 participants were assessed, and 54 participants were followed up with in the second 6-month period. There were 18 participants with missing values at 12 months. We analyzed detailed data uploaded to the mobile app Switch (Huraypositive Inc) and developed a novel UUS. Participants who received TMC were divided into 2 groups based on UUS for the first 3 months. We evaluated the relationship between UUS as an index of patient engagement and glycemic control for diabetes management.

### Tailored Mobile Coaching (TMC) System and the Switch App

The TMC system is a medical service to support diabetes self-management through bidirectional communication between health care providers and patients by sharing data uploaded to the mobile app Switch without any additional equipment for data transmission or a web portal for users. Users of the mobile app could upload measurement data such as self-monitoring of blood glucose, blood pressure, and body weight, along with their lifestyle, including dietary records, physical activities, and medical information. Care managers sent messages to provide appropriate educational information to patients. Participants received regular mobile messages and were allowed to communicate with providers via the Switch app. Care managers analyzed the transmitted records and sent messages on the secured website twice a week. The message content included notifications for behavioral recommendations, diabetes education, and individualized advice. At any time, users could check their data by logging into the Switch app, where they could obtain information on diabetes and other metabolic diseases. Details about the TMC system and Switch app have been described in the previous study [18].

### UUS (User Utility Score)

UUS was calculated as the sum of the scores for 4 core components: frequency (days) of self-monitoring of blood glucose testing, dietary and exercise records, and message reading rate (percent). We divided the data of each component for the first 3 months into tertiles: first (T1), second (T2), and third tertiles (T3) that were 0-33, 34-75, and 76-91 days for self-monitoring of blood glucose; 0-3, 4-30, and 31-91 days for dietary records; 0-37, 38-81, and 82-91 days for exercise records; and 0%-73%, 74%-97%, and 98%-100% for message reading rates, respectively. T1, T2, and T3 were scored as 0, 1, and 2 points, respectively, and the range of UUS was 0-8.

To validate UUS accuracy using the current dataset, we used another dataset from an outpatient clinic at Kangbuk Samsung Hospital from June 2012 to March 2013 [23]. The prospective clinical study evaluated the effectiveness of mobile health-based diabetes self-management [23]. The participants from the intervention group (n=39) were used as a sample of the training set. We found that UUS was associated with a change in HbA_1c_ at 6 months; the regression coefficient was –0.078 (SD 0.037; *P*=.04).

### Measurements

The primary outcome was changes in HbA_1c_ over the 12-month study period. The secondary outcomes were diabetes-related health outcomes and diabetes self-management. On the first visit, participants completed a self-administered questionnaire regarding demographic characteristics, social history, and other medical conditions. Smoking and drinking habits were categorized as noncurrent or current. Body mass index (BMI) was calculated as weight in kilograms divided by the square of height in meters. Blood pressure was measured in a seated position after 5 minutes of rest. Blood samples were obtained after overnight fasting to measure HbA_1c_, total cholesterol, triglycerides, high-density lipoprotein (HDL) cholesterol, and low-density lipoprotein (LDL) cholesterol. The Korean version of the Summary of Diabetes Self-Care Activities (SDSCA) questionnaire [24] and the Korean version of the Appraisal of Diabetes Scale (ADS) [25] were applied to evaluate diabetes self-management. The SDSCA includes items assessing diet (general and specific), exercise, blood glucose testing, foot care, and smoking over the past week; higher scores indicate better self-care behaviors [26]. The ADS is a stable measure of diabetes-related appraisal, with a smaller total score indicating a more positive appraisal [27]. Clinic or laboratory tests were repeated at baseline, 3, 6, and 12 months. A self-administered questionnaire was obtained at baseline and at 6- and 12-month follow-up evaluations.

### Statistical Analyses

Participants were divided into 2 groups according to the median value of UUS. The study outcomes of both groups were compared using the Student *t* test for continuous variables and a chi-square test for categorical variables. Data were expressed as a mean and standard deviation or as a number (proportion). Repeated-measures analyses of variance (ANOVA) were used to monitor differences in HbA_1c_ between the 2 groups over a 12-month period. In cases of missing follow-up visit (12 months) data, the last observation carried forward (LOCF) imputation method was used. Exploratory data analysis is used to investigate changes in HbA_1c_ from baseline at 3, 6, and 12 months for both groups. To assess an association of UUS with changes in HbA_1c_, multivariable linear regression analyses were used. Model 1 was adjusted for age and sex. Model 2 was adjusted for age, sex, BMI, systolic blood pressure, LDL, HbA_1c_ at baseline, and diabetes duration. Model 3 was adjusted for age, sex, BMI, systolic blood pressure, LDL, HbA_1c_ at baseline, diabetes duration, cigarette smoking, alcohol consumption, and ADS. The Bonferroni correction was then used to perform multiple comparisons between the 3 points of time. To identify the decrease amount in HbA_1c_, the reduction rate of HbA_1c_ was evaluated. The HbA_1c_ reduction rate was equal to the difference in HbA_1c_ divided by baseline HbA_1c_ value times 100%. Linear regression analysis was performed to test the relationship between the HbA_1c_ reduction rate and UUS. A *P* value <.05 was considered statistically signiﬁcant. All data were analyzed using SPSS (version 18.0; IBM Corp).

### Prior Presentation and Data Availability

These data were presented at the American Diabetes Association 77th Scientific Session. The data used or analyzed during this study are available from the Samsung Fire and Marine Insurance Company; however, restrictions apply to the availability of these data, which were used under the license of this study. Data are available from the authors upon reasonable request and with the permission of Samsung Fire and Marine Insurance Company.

## Results

### Participant Characteristics 

Participants were divided into 2 groups, UUS:0-4 and UUS:5-8, based on the UUS for the first 3 months. At 3 and 6 months, 72 participants were assessed: 38 participants in the UUS:0-4 group and 34 participants in the UUS:5-8 group. At 12 months, 54 participants were followed up with: 23 participants in the UUS:0-4 group and 31 participants in the UUS:5-8 group. Table 1 shows the baseline characteristics of the 2 groups. There were no significant differences between groups with regard to age, sex, BMI, blood pressure, HbA_1c_, LDL cholesterol levels, diabetes duration, cigarette smoking, alcohol consumption, and SDSCA and ADS scores. The mean HbA_1c_ level was 8.07% (SD 1.23%; 65 mmol/mol) in the UUS:0-4 group and 8.20% (SD 1.69%; 66 mmol/mol) in the UUS:5-8 group (*P*=.69).

**Table 1:** Baseline characteristics of participants divided into 2 groups based on user utility scores (UUS; n=72).

Characteristic		UUS:0-4 (n=38)	UUS:5-8 (n=34)	*P* value^a^	
Age in years, mean (SD)		50.58 (8.52)	52.38 (7.13)	.34	
**Gender, n (%)**					.40
	Male	21 (53.8)	21 (63.6)		
	Female	17 (46.2)	13 (36.4)		
Body mass index (kg/m^2^), mean (SD)		26.34 (3.19)	25.71 (3.42)	.42	
Systolic blood pressure (mmHg), mean (SD)		137.29 (16.54)	136.88 (15.50)	.92	
Diastolic blood pressure (mmHg), mean (SD)		87.34 (11.74)	86.68 (8.73)	.79	
HbA_1c_^b^ %, mean (SD)		8.07 (1.23)	8.20 (1.69)	.69	
Total cholesterol (mg/dL), mean (SD)		165.18 (25.22)	175.52 (39.05)	.20	
Triglyceride (mg/dL), mean (SD)		153.31 (61.55)	139.73 (60.75)	.35	
HDL cholesterol (mg/dL), mean (SD)		46.05 (9.12)	48.85 (13.29)	.30	
LDL cholesterol (mg/dL), mean (SD)		88.40 (24.81)	98.54 (34.52)	.17	
Current smoker, n (%)		13 (33.3)	6 (18.2)	.15	
Current alcohol drinker, n (%)		15 (38.5)	15 (45.5)	.55	
Diabetes duration in years, mean (SD)		7.28 (3.71)	7.23 (6.49)	.81	
Insulin injection, n (%)		8 (20.5)	8 (24.2)	.70	
Antihypertensive medication, n (%)		16 (41)	11 (33.3)	.50	
Antidyslipidemic medication, n (%)		24 (61.5)	14 (42.4)	.11	
**Summary of Diabetes** **Self-Care Activities** **(SDSCA) questionnaire, mean (SD)**					
	Diet total	10.47 (5.47)	11.68 (6.62)	.40	
	Exercise	5.82 (4.13)	6.26 (3.86)	.64	
	Blood glucose testing	3.00 (4.23)	4.94 (5.27)	.09	
	Foot care	3.58 (3.94)	3.09 (3.53)	.58	
Appraisal of Diabetes Scale (ADS) total, mean (SD)		19.76 (4.27)	19.18 (4.54)	.57	

^a^*P* values were derived from the Student *t* test or Pearson chi-square test.

^b^HbA_1c_: glycated hemoglobin.

### UUS and Changes in HbA_1c_

**Figure 1:**
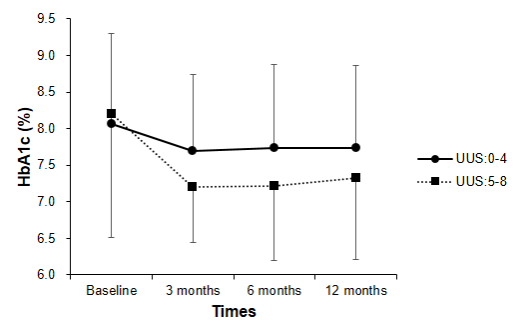
Glycated hemoglobin (HbA_1c_) levels for the 12-month study period in the UUS:0-4 group and UUS:5-8 group. Repeated-measures ANOVA revealed significant differences between the groups over 12 months (*P*=.011). The last observation carried forward (LOCF) imputation method was used. UUS: user utility score.

Figure 1 depicts mean HbA_1c_ levels for the 12-month study period in the UUS:0-4 group and UUS:5-8 group. Significant differences were observed in the improvement of HbA_1c_ within each group (*P*<.001) and between groups (*P=*.011) by repeated-measures ANOVA. The UUS:5-8 group was significantly reduced compared with the UUS:0-4 group at 3, 6, and 12 months in intention-to-treat analyses (LOCF). Table 2 shows changes in HbA_1c_ levels from baseline at 3, 6, and 12 months for both groups. At 12 months, the mean change in HbA_1c_ was –0.92 (SD 1.24%) [–10.1 (SD 13.6) mmol/mol] in the UUS:5-8 group, compared with –0.33 (SD 0.80%) [–3.63 (SD 8.8) mmol/mol] in the UUS:0**-**4 group (*P*=.049). Reductions in mean HbA_1c_ levels were greater in the UUS:5-8 group than in the UUS:0-4 group at 3 months [–1.0 (SD 1.40%) vs –0.37 (SD 0.73%); *P*=.02] and at 6 months [–0.99 (SD 1.09%) vs –0.32 (SD 1.08%); *P*=.01].

**Table 2:** Changes from baseline in glycated hemoglobin (HbA_1c_) at 3, 6, and 12 months in the UUS:0-4 group and UUS:5-8 group.

HbA_1c_ at each time point		UUS^a^:0-4	UUS:5-8	*P* value^b^
**3 months from baseline (n=72)**				
	Participants, n (%)	38 (53)	34 (47)	
	HbA_1c_ %, mean (SD)	–0.37 (0.73)	–1.0 (1.40)	.018
**6 months from baseline (n=72)**				
	Participants, n (%)	38 (53)	34 (47)	
	HbA_1c_ %, mean (SD)	–0.32 (1.08)	–0.99 (1.09)	.013
**12 months from baseline (n=54)**				
	Participants, n (%)	23 (43)	31 (57)	
	HbA_1c_ %, mean (SD)	–0.33 (0.80)	–0.92 (1.24)	.049

^a^UUS: user utility score.

^b^*P* values were derived from the Student *t*-test.

Table 3 shows the association of UUS for the first 3 months with changes in HbA_1c_ at 3, 6, and 12 months. Simple linear regression analysis revealed a significant association of UUS with changes in HbA_1c_ at 3, 6, and 12 months in the LOCF analysis. In multivariable linear regression analyses, after adjustment for age, sex, BMI, systolic blood pressure, LDL cholesterol, HbA_1c_ at baseline, diabetes duration, cigarette smoking, alcohol consumption, and ADS score (model 3), UUS was significantly associated with changes in HbA_1c_ at 3, 6, and 12 months; the regression coefficients were –0.113 (SD 0.040; *P*=.006), –0.143 (SD 0.045; *P*=.002), and –0.136 (SD 0.052; *P*=.011), respectively. In model 3, the result was still significant under the Bonferroni adjustment for multiple comparisons between 3 points of time. In addition, UUS was inversely associated with reduction rates of HbA_1c_ at 3, 6, and 12 months; the regression coefficients were –2.70 (SD 1.24; *P*=.03), –3.35 (SD 1.37; *P*=.02), and –3.19 (SD 1.59; *P*=.049), respectively (Table 4).

**Table 3:** Association of user utility score (UUS) with change in glycated hemoglobin (HbA_1c_) by multivariable linear regression analyses.

UUS	3 months from baseline			6 months from baseline			12 months from baseline^a^	
	β (SE^b^)	*P* value^c^	β (SE)		*P* value	β (SE)		*P* value
Crude	–0.121 (0.055)	.031	–0.125 (0.054)		.023	–0.124 (0.048)		.011
Model 1^d^	–0.119 (0.057)	.042	–0.127 (0.057)		.028	–0.128 (0.050)		.012
Model 2^e^	–0.100 (0.037)	.009	–0.109 (0.043)		.014	–0.108 (0.051)		.038
Model 3^f^	–0.113 (0.040)	.006	–0.143 (0.045)		.002	–0.136 (0.052)		.011

^a^The last observation carried forward (LOCF) imputation method was used.

^b^SE: standard error.

^c^*P* values in multiple regression models are significant at *P*<.05. *P* values after Bonferroni correction are significant at *P*<.016.

^d^Model 1 was adjusted for age and sex.

^e^Model 2 was adjusted for age, sex, BMI, systolic blood pressure, LDL cholesterol, HbA_1c_ at baseline, and diabetes duration.

^f^Model 3 was adjusted for age, sex, BMI, systolic blood pressure, LDL cholesterol, HbA_1c_ at baseline, diabetes duration, cigarette smoking, alcohol consumption, and the Appraisal of Diabetes Scale (ADS) score.

**Table 4:** Association of user utility score (UUS) with glycated hemoglobin (HbA_1c_) reduction rate by linear regression analyses.

UUS at each time point	β (SE^a^)	*P* value^b^
3 months from baseline	–2.70 (1.24)	.033
6 months from baseline	–3.35 (1.37)	.017
12 months from baseline^c^	–3.19 (1.59)	.049

^a^SE: standard error.

^b^Regression coefficients (β) and *P* values were derived from linear regression analysis.

^c^The last observation carried forward (LOCF) imputation method was used.

### UUS and Health Outcomes

Table 5 shows changes in health outcomes from baseline at 3, 6, and 12 months in the UUS:0-4 group and UUS:5-8 group. At 3 months, HDL cholesterol levels between the UUS:0-4 and UUS:5-8 groups were –2.03 (SD 7.02) mg/dL and 1.48 (SD 7.33) mg/dL, respectively (*P*=.04). There were no significant differences in the changes in BMI, systolic blood pressure, HDL cholesterol, and SDSCA and ADS scores, after Bonferroni adjustment, between the 2 groups at 3, 6, and 12 months. There was a suggestion that the high UUS was more beneficial for HDL cholesterol, with the *P* value indicating a significant difference on the basis of the conventional threshold for significance but not the Bonferroni-adjusted threshold.

**Table 5:** Changes in biochemical parameters and questionnaires over 12 months in the UUS:0-4 group and UUS:5-8 group.

Variable, mean (SD)		3 months from baseline (n=72)					6 months from baseline (n=72)				12 months from baseline (n=54)		
		UUS:0-4 (n=38)		UUS:5-8 (n=34)	*P* value^a^	UUS:0-4 (n=38)		UUS:5-8 (n=34)	*P* value	UUS:0-4 (n=23)		UUS:5-8 (n=31)	*P* value
Body mass index, kg/m^2^		–0.25 (0.67)		–0.42 (0.85)	.36	–0.41 (0.82)		–0.32 (0.93)	.68	0.01 (0.92)		0.04 (1.16)	.89
Systolic blood pressure, mmHg		–15.45 (16.82)		–14.38 (16.14)	.79	–17.29 (16.27)		–16.29 (14.15)	.78	–8.01 (16.77)		–7.32 (13.82)	.87
Diastolic blood pressure, mmHg		–7.34 (10.37)		–7.26 (8.87)	.97	–9.84 (10.22)		–6.82 (8.46)	.18	–1.91 (9.65)		–2.97 (7.61)	.66
Total cholesterol, mg/dL		3.56 (32.44)		10.82 (26.50)	.31	–1.15 (31.80)		3.79 (32.15)	.52	6.30 (43.66)		–1.74 (38.18)	.53
Triglyceride, mg/dL		3.87 (72.91)		–17.76 (42.42)	.14	–5.03 (78.00)		–8.30 (51.79)	.84	25.39 (90.86)		–6.65 (60.32)	.14
HDL cholesterol, mg/dL		–2.03 (7.02)		1.48 (7.33)	.04	0.28 (6.48)		2.61 (7.37)	.16	0.43 (7.09)		4.61 (8.55)	.06
LDL cholesterol, mg/dL		4.75 (29.84)		12.78 (24.80)	.22	–0.49 (28.75)		2.85 (28.82)	.63	1.33 (38.63)		–5.07 (35.29)	.53
**Summary of Diabetes Self-care Activities (SDSCA) questionnaire, mean (SD)**													
	Diet total		N/A^b^	N/A	N/A	1.24 (7.05)		1.47 (7.82)	.894	2.23 (6.87)		1.35 (5.35)	.61
	Exercise		N/A	N/A	N/A	0.56 (3.79)		0.74 (3.06)	.83	0.58 (2.69)		1.36 (3.14)	.34
	Blood glucose testing		N/A	N/A	N/A	2.29 (5.56)		4.15 (6.11)	.38	0.32 (4.94)		1.13 (5.92)	.60
	Foot care		N/A	N/A	N/A	3.66 (5.23)		2.79 (5.47)	.50	3.59 (5.79)		2.97 (4.35)	.66
Appraisal of Diabetes Scale (ADS) total		N/A		N/A	N/A	–0.79 (4.82)		–1.5 (4.97)	.54	–1.41 (4.55)		–1.81 (3.75)	.73

^a^*P* values were derived from the Student *t* test.

^b^N/A: not applicable.

## Discussion

### Principal Findings

In this study, we found that UUS for the first 3 months was associated with changes in HbA_1c_ during a 12-month follow-up period. When we divided participants into 2 groups based on UUS for the first 3 months, the high UUS group resulted in greater decreases in HbA_1c_ over 12 months compared with the low UUS group. Multivariable linear regression analyses revealed that UUS for the first 3 months was significantly inversely associated with changes in HbA_1c_ at 3, 6, and 12 months. These results indicated that initial active engagement for the first 3 months with a mobile health application was associated with improved glycemic control over the whole study period.

Patient engagement with a mobile health application could be a significant factor contributing to diabetes self-management [28,29]. Research showed that initial engagement with a mobile health application is closely related to long-term engagement [30]. We previously reported that initial active engagement was significantly correlated with improved glycemic control [31]. In this study, UUS gradually decreased over 12 months but was consistently higher in the initial high UUS group than in the low UUS group. In addition, more participants were lost to follow-up in the low UUS group (15/38, 39%) than in the high UUS group (3/34, 9%) at 12 months. High UUS in the beginning was critical. We found that initial active engagement could predict improved glycemic control during a 12-month follow-up period. Therefore, initial strategies to enhance patient engagement from the beginning in the low UUS group are necessary.

We developed the UUS as a tool to measure user utility by analyzing and scoring uploaded data, including blood glucose testing, dietary and exercise records, and message reading. The results of this study reinforce findings from previous mobile health investigations that have shown the benefits of lifestyle interventions with appropriate blood glucose testing, adoption of a healthy diet and physical activity, and reading of text messages on diabetes outcomes [31-36]. Meanwhile, patient engagement is related to other factors such as medications, foot care, and changes in weight [37]. To quantify patient engagement, scoring systems should take into account that each factor in UUS varies in priority and importance. Our findings will act as a cornerstone for other studies exploring effective UUS components and the optimal threshold of each component for predicting improved health outcomes.

In addition, we found no difference in baseline HbA_1c_, BMI, blood pressure, HDL cholesterol, LDL cholesterol, SDSCA, and ADS to assess diabetes self-management between groups divided according to UUS. There was no relationship of UUS with blood pressure, lipid profile, or diabetes self-management. Although the high UUS group achieved greater improvement in HDL cholesterol at 3 months than the low UUS group, this difference was not statistically significant. Regular exercise increases HDL cholesterol levels [38,39], and so it would be interesting to evaluate the association between patient engagement and HDL cholesterol. Further randomized controlled trials are needed to investigate the relationship between patient engagement and health outcomes among patients with type 2 diabetes. However, UUS was not related to cardiovascular risk factors such as LDL, blood pressure, and BMI. The results support the concept that UUS could be a useful tool for predicting improved glycemic control in diabetes management using the TMC system.

Chronic diseases such as diabetes that require ongoing medical care can benefit from the integration of digital health technology-based tools [16]. Digital health technology in diabetes care offers the opportunity to track and visualize data regarding parameters such as blood glucose testing, dietary habits, physical activity, and text messages and has been promoted to support self-management and facilitate lifestyle changes [11,40,41]. We found that UUS with behavioral components was correlated with changes in HbA_1c_ in a 12-month follow-up evaluation (r=–0.136, *P*=.01). According to the SDSCA, the frequency of blood glucose testing was higher in patients with a high UUS score than in those with a low UUS score (*P*=.09). This seems to be the most important contributing factor to the results of this study. Interestingly, however, individual components of UUS were not correlated with changes in HbA_1c_. This study did not show that patient engagement was not associated with each individual behavioral component.

Patient engagement assessment tools could be useful for evaluating their own diabetes self-management [42]. Remmers et al [43] examined the association of patient activation measure (PAM) scores with health outcomes among patients with diabetes and found that PAM scores could be used to identify patients at risk for poorer health outcomes. Previous studies that found differences between patients who engage and those who do not engage in digital health interventions demonstrated the importance of patient engagement to glycemic control [39]. Our results support the idea that although digital technology will not provide a solution for everyone, the use of mobile health technology tools, when applied appropriately, could improve the health outcomes of patients with diabetes [44]. Moreover, long-term management is critical because people usually participate actively in the beginning, but their interest disappears. We suggest that the optimal use of UUS should be individualized based on the clinical needs of individual patients and the requirements of care providers. Further investigation regarding how to motivate participants toward engaging in this digital health system is needed.

### Limitations

There were several limitations to the study. First, although the relationship between UUS and glycemic control was statistically significant, the post-hoc analysis study design was one limitation. It is important to adjust for confounding variables, potentially influencing patient outcomes. The use of digital health tools may be influenced by education level and social deprivation, among other factors. Although these variables were not included as covariates in the model, any response bias is likely minimal. Second, UUS consisted of only 4 behavioral components and was calculated retrospectively. However, we used a holdout set for validation and introduced UUS to project user participation and effects on glycemic control. Finally, the study had a small sample size, and values for 25% (18/72) of participants were missing at 12 months. Regression analysis of the association between UUS and HbA_1c_ was performed using the LOCF approach to examine trends over time, rather than focusing simply on the endpoint. This imputation might lead to biased results. However, for the comparison of HbA_1c_ between the UUS groups, data were analyzed without applying LOCF, which revealed a significant group difference. Larger prospective long-term studies are needed to assess the UUS's utility in a real-world setting.

### Conclusions

In this study, we developed the UUS as a patient engagement measure with behavioral components from an individual perspective. UUS in the beginning was associated with changes in HbA_1c_ over the study period of the TMC system and could be a useful tool for predicting improved glycemic control in diabetes self-management through mobile health interventions. Our results provide insight into the importance of patient engagement in mobile diabetes intervention, and further studies to explore the optimal measure of patient engagement for diabetes management are needed.

## Abbreviations

ADS: Appraisal of Diabetes Scale
BMI: body mass index
HbA_1c_: glycated hemoglobin
HDL: high-density lipoprotein
LDL: low-density lipoprotein
LOCF: last observation carried forward
PAM: patient activation measure
SDSCA: Summary of Diabetes Self-Care Activities questionnaire
TMC: tailored mobile coaching
UUS: user utility score
